# Characterization of Two Cases of Congenital Dyserythropoietic Anemia Type I Shed Light on the Uncharacterized C15orf41 Protein

**DOI:** 10.3389/fphys.2019.00621

**Published:** 2019-05-22

**Authors:** Roberta Russo, Roberta Marra, Immacolata Andolfo, Gianluca De Rosa, Barbara Eleni Rosato, Francesco Manna, Antonella Gambale, Maddalena Raia, Sule Unal, Susanna Barella, Achille Iolascon

**Affiliations:** ^1^Dipartimento di Medicina Molecolare e Biotecnologie Mediche, Università degli Studi di Napoli Federico II, Naples, Italy; ^2^CEINGE Biotecnologie Avanzate, Naples, Italy; ^3^Division of Pediatric Hematology, Hacettepe University, Ankara, Turkey; ^4^SSD Talassemie, Anemie Rare e Dismetabolismi del Ferro, Ospedale Pediatrico Microcitemico Antonio Cao, Azienda Ospedaliera Brotzu, Cagliari, Italy

**Keywords:** CDA (I–III), *C15ORF41*, functional characterization of proteins, genetic testing, anemia

## Abstract

CDA type I is a rare hereditary anemia, characterized by relative reticulocytopenia, and congenital anomalies. It is caused by biallelic mutations in one of the two genes: (i) *CDAN1*, encoding Codanin-1, which is implicated in nucleosome assembly and disassembly; (ii) *C15orf41*, which is predicted to encode a divalent metal ion-dependent restriction endonuclease with a yet unknown function. We described two cases of CDA type I, identifying the novel variant, Y94S, in the DNA binding domain of C15orf41, and the H230P mutation in the nuclease domain of the protein. We first analyzed the gene expression and the localization of C15orf41. We demonstrated that *C15orf41* and *CDAN1* gene expression is tightly correlated, suggesting a shared mechanism of regulation between the two genes. Moreover, we functionally characterized the two variants, establishing that the H230P leads to reduced gene expression and protein level, while Y94S induces a slight decrease of expression. We demonstrated that C15orf41 endogenous protein exhibits nuclear and cytosolic localization, being mostly in the nucleus. However, no altered nuclear-cytosolic compartmentalization of mutated C15orf41 was observed. Both mutants accounted for impaired erythroid differentiation in K562 cells, and H230P mutant also exhibits an increased S-phase of the cell cycle in these cells. Our functional characterization demonstrated that the two variants have different effects on the stability of the mutated mRNA, but both resulted in impaired erythroid maturation, suggesting the block of cell cycle dynamics as a putative pathogenic mechanism for C15orf41-related CDA I.

## Introduction

Congenital dyserythropoietic anemias (CDAs) are hereditary diseases, belonging to the bone marrow (BM) failure syndromes, which embrace a heterogeneous set of rare hereditary anemias that result from impaired erythropoiesis and various kinds of abnormalities during late stages of erythropoiesis (Gambale et al., 2016). Among them, CDA type I (CDA I) is characterized by anemia of variable degree, generally macrocytic, relative reticulocytopenia, and congenital anomalies, such as syndactyly, chest deformity, and short stature. The original classification system for CDAs was based on specific erythroblasts morphological abnormalities on BM light microscopy (Roy and Babbs, 2019). The morphological pathognomonic feature of CDA I is the presence of thin chromatin bridges between the nuclei pairs of erythroblasts. On electron microscopy, heterochromatin is denser than normal, and forms demarcated clumps with small translucent vacuoles, giving rise to the metaphor of “Swiss cheese appearance” (Kellermann et al., 2010; Roy and Babbs, 2019).

CDA I is inherited as an autosomal recessive disorder caused by mutations in two different loci, *CDAN1* and *C15orf41*, which account for the 90% of CDA I cases. *CDAN1* (chr15q15.2) was the first gene in which pathogenic variants causative of CDA type I (OMIM # 224120) were identified (Dgany et al., 2002). It encodes a ubiquitously expressed and cell-cycle regulated protein, Codanin-1 (Noy-Lotan et al., 2009), which acts in nucleosome assembly and disassembly through the formation of the cytosolic Asf1-H3-H4-importin-4 complex. Codanin-1 binds directly to Asf1 via a conserved B-domain, implying a mutually exclusive interaction with the chromatin assembly factor 1 (CAF-1) and HIRA. Previous studies on osteosarcoma U-2-OS cells silenced for Codanin-1 showed accelerated DNA replication rate and increased levels of chromatin-bound Asf1, suggesting that Codanin-1 guards a limiting step in chromatin replication (Ask et al., 2012). More recently, *C15orf41* (chr15q14) was discovered as the second locus associated with CDA I (OMIM # 615631). It is an uncharacterized gene that is predicted to encode a divalent metal-ion dependent restriction endonuclease with homology to the Holliday junction resolvases (Babbs et al., 2013). It was suggested that C15orf41-encoded protein, similarly to Codanin-1, interacts with Asf1b (Ewing et al., 2007), supporting the hypothesis that both C15orf41 and Codanin-1 could interplay during DNA replication and chromatin assembly (Gambale et al., 2016).

To date, only five *C15orf41* variants have been reported (Babbs et al., 2013; Palmblad et al., 2018; Russo et al., 2018). We herein described two cases of *C15orf41*-CDA I carrying the aminoacidic substitutions p.Tyr94Ser and p.His230Pro that are located in the two different domains of the C15orf41 protein. Our functional characterization demonstrated that the two variants have different effects on the stability of the mutated mRNA. However, both mutations account for impaired erythroid maturation. This study improves the current understanding of the role of this uncharacterized protein in both the physiological conditions and the pathogenic mechanism of the disease.

## Materials and Methods

### Patients and Genetic Testing

The diagnosis of CDA I was based on history, clinical findings, laboratory data, morphological analysis of both peripheral blood and marrow smears, and genetic testing.

Local university ethical committees approved both the DNA sampling and the collection of patients’ data from Medical Genetics Ambulatory in Naples (University Federico II, DAIMedLab).

Written informed consent was obtained from the patients for the participation in the study and the publication of the case report.

Genomic DNA preparation and mutational screening for *CDAN1, SEC23B*, and *C15orf41* genes by direct sequencing were performed as previously described (Russo et al., 2013). High-throughput sequencing by the custom multi-gene panel for hereditary anemias was performed as described (Russo et al., 2018).

The pathogenicity of the novel exonic variants has been evaluated by InterVar, a bioinformatics software tool for clinical interpretation of genetic variants based on the ACMG/AMP 2015 guideline^1^. Mainly, the pathogenicity of each variant was assessed by gathering evidence from various sources: population data, computational and predictive data, functional data, localization of the variant in a mutational hotspot and critical and well-established functional domain, and segregation data (Richards et al., 2015; Russo et al., 2018).

### Cloning and Site Direct Mutagenesis

cDNA encoding full-length wild-type (WT) *C15orf41* sequence was cloned in the pCMV-Tag1 vector for mammalian cell expression (Invitrogen) in the BglII and XhoI sites, to obtain an N-terminal tagged protein with FLAG. The point mutations c.281A > C, p.Tyr94Ser (Y94S) and c.689A > C, p.His230Pro (H230P) were introduced into the pCMV-Tag1 vector by using a QuikChange site-directed mutagenesis kit (Stratagene) (Russo et al., 2017). The coding sequence was sequenced after mutagenesis.

### Cell Cultures, Transfections, and Stable Clones Production

Hek-293, HepG2, HuH7, MG-63, HEL, and K562 cells were obtained from American Type Culture Collection (ATCC, Manassas, VA, United States). Cells were maintained in Dulbecco’s modified Eagle medium (DMEM) (Invitrogen) or RPMI 1640 medium (Invitrogen) supplemented with 10% fetal bovine serum (Invitrogen), 100 U/mL penicillin (Invitrogen), and 100 mg/mL streptomycin (Invitrogen) in a humidified 5% CO_2_ atmosphere at 37°C, according to the manufacturer’s instructions. Hek-293 cells (400 × 10^3^) were transfected with pCMV-Tag1-C15orf41 plasmids (2.5 μg/well) using the DNA Transfection Reagent (TransFectin Lipid Reagent, Bio-Rad) according to the manufacturer’s procedures. Cells were collected 16, 24, and 48 h after the transfection to perform RNA and protein extractions. For generating K562 stably over-expressing *C15ORF41* gene, 10^6^ cells were transfected with pCMV-Tag1-C15orf41 plasmids using Hily Max DNA Transfection Reagent (Dojindo Laboratories). After 48 h, G418 (0.6 mg/mL) was added as a selection marker. Clones were generated according to the limiting dilution method (see Supplementary Material for further details).

### Erythroid Differentiation and Flow Cytometry

Erythroid differentiation of K562-*C15orf41* stable clones (2 × 10^5^/mL) was performed adding 50 μM hemin (Sigma) to the culture medium, after 24 h of starvation (Andolfo et al., 2010). Cells were collected before hemin addition (0 days) and two days after hemin addition (2 days). For cell cycle analysis, K562 stable clones were harvested by centrifugation, resuspended in PBS containing 3.75% Nonidet P-40, 100 μg/ml RNase A and 40 μg/ml propidium iodide, and incubated at room temperature for 3 h in the dark. The cell antigen profile was analyzed by flow cytometry through evaluation of CD71 (proerythroblasts) and CD235a (proerythroblasts and orthochromatic erythroblasts). Samples were analyzed on a FACS flow cytometer (Becton Dickinson Immunocytometry Systems, BDIS).

### Gene Expression Analysis

Total RNA was extracted either from peripheral blood leukocytes (PBLs), reticulocytes and from cell lines using TRIzol reagent (Life Technologies). Synthesis of cDNA from total RNA (2 μg) was performed using SensiFAST^TM^ cDNA Synthesis Kit (Bioline). Quantitative RT-PCR (qRT-PCR) using Power SYBR Green PCR Master Mix (Applied Biosystems) was performed on Applied Biosystems 7900HT Sequence Detection System using standard cycling conditions. *β-actin* was used as internal control, while the *Neomycin* resistance gene was used as a control of transfection efficiency for K562 stable clones. Relative gene expression was calculated by using the 2^−ΔCt^ method, as described (Russo et al., 2013).

### Subcellular Fractionation and Western Blotting

Proteins were extracted from cell lines using RIPA lysis buffer containing protease inhibitor cocktail (1×). Subcellular fractionation in nuclear and cytoplasmic proteins was performed using NE-PER^TM^ Nuclear and Cytoplasmic Extraction Reagents (Thermo Fisher Scientific^TM^). Equal amounts of protein from each lysate, as determined by a Bradford assay, were subjected to 12% sodium dodecyl sulfate-polyacrylamide gel electrophoresis (SDS-PAGE), and blotted onto polyvinylidene difluoride membranes (Biorad). Detection was performed with mouse anti-FLAG antibody (1:1000) (Sigma-Aldrich) and rabbit anti-C15orf41 (1:500) (Atlas Antibodies HPA061023). Since this antibody was recommended for immunofluorescence (IF) we tested its specificity for western blotting (WB) by using C15orf41 over-expression cells as a positive control (Bordeaux et al., 2010) (Supplementary Figure S1).

Mouse anti-TBP (TATA Binding Protein) (1:1000) (Sigma-Aldrich) and mouse anti-α-TUBULIN (1:5000) (Abcam) were used as a control for equal loading for cytosolic and nuclear proteins’ extracts, respectively. Mouse anti-β-actin (1:12000) (Sigma-Aldrich) was used as a loading control for total proteins’ extracts. Labeled bands were visualized and densitometric analysis performed with the BioRad Chemidoc using Quantity One software (BioRad) to obtain an integrated optical density (OD) value.

### Immunofluorescence Analysis

For IF analysis 3 × 10^5^ cells were fixed for 10 min in 4% Paraformaldehyde (PFA, Sigma) and washed in 50 mM PBS/NH4Cl (Sigma-Aldrich, Milan, Italy). After washing in PBS 1×, cells were allowed on 35 mm IBIDI μ-Dishes (Ibidi GmbH, Martinsried, Germany) coated with 0.05% poly-L-lysine (Sigma-Aldrich, Milan, Italy) to adhere. Permeabilization was performed with 0.2% Triton/PBS, followed by blocking with 1% BSA/PBS. The seeded cells were immunologically stained with rabbit anti-C15orf41 antibody (1:25) (Atlas Antibodies HPA061023), mouse anti-NUCLEOPHOSMIN (1:200), and secondary antibodies (1:200) (Alexa Fluor 546 anti-rabbit, Life Technologies and Alexa Fluor 488 anti-mouse). Nuclei were stained with 1 μg/ml DRAQ5 in PBS for 15 min at room temperature. Cells were preserved in PBS 1× and imaged using a LEICA TCS SP8 meta confocal microscope, equipped with an oil immersion plan Apochromat 63× objective 1.4 NA. The following settings were used: Green channel excitation of Alexa488 by the argon laser 488 nm line was detected with the 505–550 nm emission bandpass filter. Red channel excitation of Alexa546 by the Helium/Neon laser 543 nm line was detected with the 560–700 nm emission bandpass filter (using the Meta monochromator). Blue channel excitation of DRAQ5 by the blue diode laser 647 nm and emission bandpass filter.

### Statistical Analysis

Statistical significance of differences in protein and gene expression was determined using the Mann–Whitney test or Student’s *t*-test. Correlation analysis of *C15orf41* with *CDAN1* gene expression was performed by Pearson correlation test. A two-sided *p*-value < 0.05 was considered statistically significant.

For the *in silico* correlation analysis between *C15orf41* and *CDAN1* gene expression in normal hematopoietic cell subpopulations we used the dataset “Normal Hematopoietic Subgroups – (GEO ID: gse19599),” stored in the R2: Genomics Analysis and Visualization Platform^2^, a biologist-friendly, web-based genomics analysis, and visualization application.

## Results

### Clinical Cases and Genetic Testing

**FIGURE 1:**
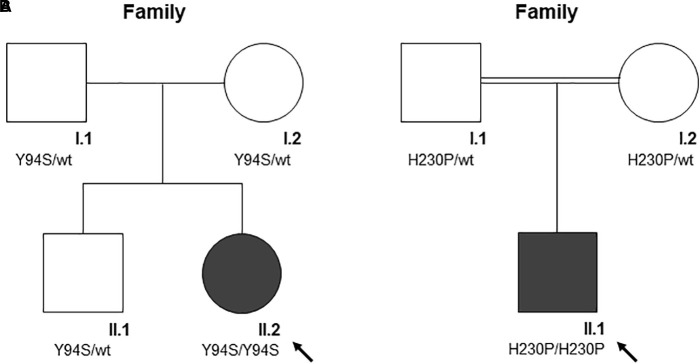
The pedigree of the two families (**A** and **B**). Square denote males, circle females, solid symbols affected persons. The black arrow indicates probands. **(A)** The pedigree of family A is shown. According to the autosomal recessive inheritance pattern, the parents of A-II.2 are heterozygous for the variant c.281A > C, p.Tyr94Ser. **(B)** The pedigree of family B is shown. According to the autosomal recessive inheritance pattern, consanguineous parents of B-II.1 are heterozygous for the variant c.689A > C, p.His230Pro.

Clinical features and genetic data of the two probands are summarized in Table 1. Case 1 (A-II.2) was a 7-years-old female, second child from healthy non-consanguineous parents of Italian origin (Sardinia). At birth, cholestatic hepatopathy, dysmorphic features (bilateral syndactyly of the IV–V toes), and severe anemia (Hb 5.5 gr/dl) were observed. Family history was not indicative of anemia. At diagnosis, the proband presented transfusion-dependent normocytic anemia with a blood transfusion frequency every 15–20 days, and low reticulocyte count (Table 1). BM analysis showed: erythroid hyperplasia with 6% of cells showing megaloblastic features, nuclear abnormalities, and nuclear/cytoplasmic maturation asynchrony; 4% of erythroblasts were bi- and tri-nucleated; the granulopoietic/erythropoietic ratio (G:E) = 0.53. A substantial percentage of erythroblasts showed inter-nuclear bridges (5%), a typical feature of CDA I. Accordingly, genetic testing for *CDAN1* was performed, but no causative variants were identified. Conversely, when we analyzed *C15orf41* gene, we observed the presence of the transversion c.281A > C in the homozygous state, resulting in a novel aminoacidic substitution p.Tyr94Ser (Y94S). It is an ultra-rare variant (rs587777101) with a minor allele frequency (MAF) C = 0.00001 in the ExAC database. In agreement with the recessive inheritance pattern, both parents were heterozygous (Figure 1A).

**Table 1:** Clinical features of the two patients enrolled in the study.

	Case 1 *(A-II.2)*	Case 2 *(B-II.1)^∗^*	Reference range^‡^
Age at diagnosis	7 years	2.4 years	–
Distal limb anomalies/ other features	Toes syndactyly	Thoracic dysplasia; short limbs	–
**Complete blood count**			
RBC (×10^6^/μL)	2.72	3.67	3.9–5.6
Hb (g/dL)	7.8	10.6	11.0–16.0
Hct (%)	22.2	31.4	33.0–45.0
MCV (fL)	81.6	85.4	70.0–91.0
MCH (pg)	20.2	28.8	23.0–33.0
MCHC (g/dL)	24.8	33.8	23.0–33.0
Retics %	5.8	1.0	0.5–2.0
Retics count (×10^3^/μL)	158000	36700	–
PLT (×10^3^/μL)	–	518.0	150.0–450.0
**Biochemical, laboratory data and iron balance**			
Total bilirubin (mg/dL)	1.90	1.46	0.2–1.2
LDH (U/L)	779	511	125.0–243.0
Ferritin (ng/mL)	825	1512	22.0–275.0
TSAT (%)	75	89	15.0–45.0
**C15ORF41 variants**			
HGVS (Coding)^a^	c.281A > C	c.689A > C	–
HGVS (Protein)^b^	p.Tyr94Ser	p.His230Pro	–
RefSeq ID	rs587777101	–	–
MAF	C = 0.00001	–	–
InterVar (evidence codes)^§^	Pathogenic (PS1, PS3, PM2, PP4)	Likely pathogenic (PS3, PM2, PP4)	–

*^∗^Patient RP0_39 described in Russo et al. (2018); ^‡^Reference ranges from AOU Federico II, University of Naples, Italy; ^a^NM_001130010; ^b^NP_001123482; ^§^ InterVar evidence scores by the website http://wintervar.wglab.org/evds.php; PS1, same amino acid change as an established pathogenic variant; PS3, well-established functional studies show a deleterious effect; PM2, absent (or at an extremely low frequency if recessive) in population databases; PP4, patient’s phenotype is highly specific for a single gene etiology; TSAT, transferrin saturation; MAF, minor allele frequency.*

Case 2 was a 2.4-years-old male, born from 3rd degree consanguineous parents of Turkish origin. At birth, recurrent pneumonia, thoracic dysplasia, and short limbs were observed. Family history was negative for anemia or jaundice. The proband presented transfusion-dependent normocytic anemia (12 transfusions/year), low reticulocyte count, growth retardation, and increased ferritin level, suggesting an iron loading condition (Table 1). No splenomegaly was observed at physical examination and abdominal echography. BM analysis showed severe megaloblastic changes and normoblasts with double or multiple nuclei, a morphological feature suggestive of CDA II. Accordingly, we firstly performed Sanger sequencing analysis for CDA II-disease gene *SEC23B*, finding no causative variants. Then, as a second-step analysis, we enrolled the patient in our multi-gene panel for hereditary anemias, identifying the transversion c.689A > C in *C15orf41* in the homozygous state, resulting in the amino acid substitution p.His230Pro (H230P), as reported (Russo et al., 2018). In agreement with the recessive inheritance pattern, both parents were heterozygous (Figure 1B).

**FIGURE 2:**
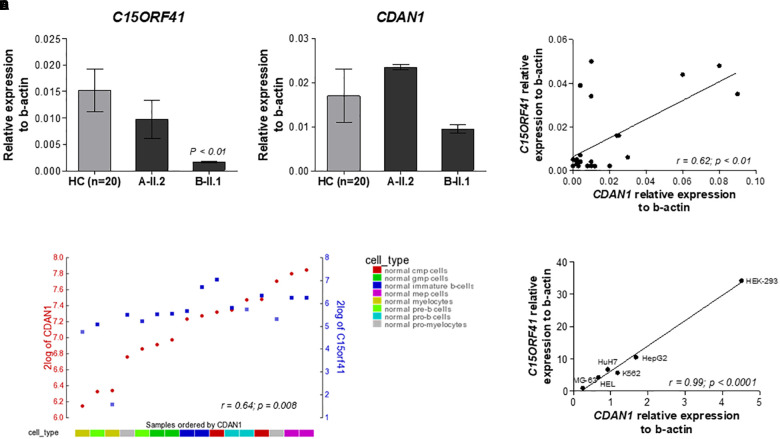
Analysis of the *C15orf41* and *CDAN1* expression. **(A)** *C15orf41* mRNA relative expression to *β-actin* of patients A-II.2, B-II.1, and HCs (*n* = 20) are shown. Data are presented as mean ± SE. *P* value by Student’s *t*-test. **(B)** *CDAN1* mRNA relative expression to *β-actin* of patients A-II.2, B-II.1 and HCs (*n* = 20) is shown. Data are presented as mean ± SE. **(C)** Correlation analysis between *C15orf41* and *CDAN1* gene expression, performed on 20 HCs and two probands, showed a direct correlation between the two genes (Pearson correlation *r* = 0.62, *p* = 0.01). **(D)** YY-plot of correlation analysis performed by R2 database to investigate the expression level of *C15orf41* and *CDAN1* genes in the expression dataset for normal flow sorted hematopoietic cell subpopulation (GEO ID: gse19599); CMP, common myeloid progenitor; GMP, granulocyte-macrophage progenitor. Pearson correlation *r* = 0.64, *p* = 0.008. **(E)** Correlation analysis between *C15orf41* and *CDAN1* expression, performed in MG-63, HEL, K562, HuH7, HepG2, and Hek-293 cell lines. MG-63, bone osteosarcoma cells; HEL, human erythroblasts; HuH7, hepatocellular carcinoma cells; HepG2, hepatocellular carcinoma cells (Pearson correlation *r* = 0.99, *p* < 0.0001).

### *C15orf41* and *CDAN1* Gene Expression Are Directly Correlated

To evaluate the effect of the two identified mutations on C15orf41 gene expression, we initially analyzed *C15orf41* expression in PBLs isolated from the two probands and healthy controls (HCs). To note, *C15orf41* is a ubiquitous gene, showing a comparable level of expression in both PBLs and reticulocytes (Supplementary Figure S2). No difference in gene expression levels of the proband A-II.2 compared to those detected in HCs was observed, suggesting that Y94S variant does not affect gene expression. Conversely, we found a marked down-regulation of *C15orf41-*H230P in the second proband B-II.1 (Figure 2A). Likewise, we saw a similar trend of *CDAN1* expression in the two patients. Notably, the proband A-II.2 did not show any alterations of *CDAN1* expression compared to those seen in HCs, while the B-II.1 proband revealed a decrease of *CDAN1* expression level, although not statistically significant (Figure 2B). Of note, a direct correlation between *C15orf41* and *CDAN1* expression genes in healthy subjects was observed (*r* = 0.62, *p* = 0.0006) (Figure 2C). We confirmed the *ex vivo* data on *C15orf41*-*CDAN1* correlation by *in silico* analysis of the expression dataset for normal hematopoietic cell subpopulations, obtained by R2 database (Figure 2D). Additionally, we achieved comparable results by gene expression profiling of different human cell lines (Hek-293, HepG2, HuH7, MG-63, HEL, and K562 cells), where a significant direct correlation between *C15orf41* and *CDAN1* expression was observed (Figure 2E).

**FIGURE 3:**
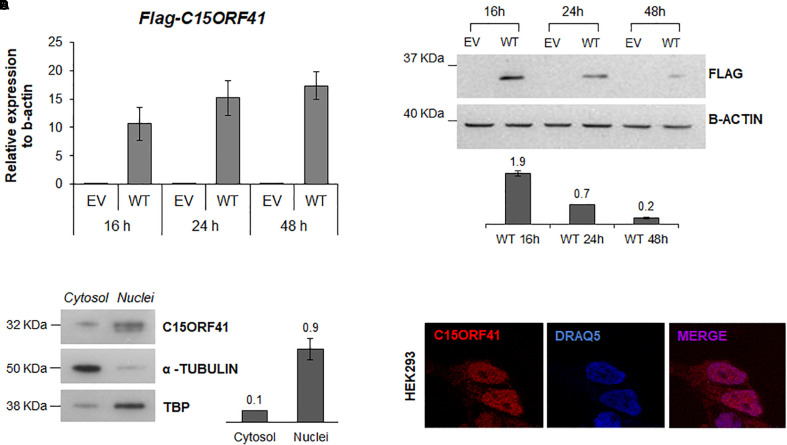
C15orf41-WT expression and subcellular localization. **(A)** The panel shows *FLAG-C15orf41* mRNA relative expression to β-actin of Hek-293 cells over-expressing pCMV-tag1-C15orf41 WT compared to those transfected with empty vector (EV) at 16, 24, and 48 h after the transfection. Data from two different transfections are presented as mean ± SD. **(B)** The panel shows WB analysis of Hek-293 cells over-expressing pCMV-tag1-C15orf41 WT compared to those transfected with EV at 16, 24, and 48 h after the transfection. β-actin is loading control. Sizes (in kDa) are on the left. The histogram shows the densitometric quantification based on β-actin amount. Data derived from two experiments are presented as mean ± SD. **(C)** WB on cytosolic and nuclear fractions of Hek-293 cells showing C15orf41 expression. TBP and α- TUBULIN are shown as a loading control of nuclear and cytosolic compartments, respectively. The histogram shows the densitometric quantification based on TBP and α- TUBULIN amounts. Data derived from three experiments are presented as mean ± SD. Sizes (in kDa) are on the left. **(D)** Immunofluorescence analysis of Hek-293 cells is shown. Rabbit anti-C15orf41 antibody was used to stain C15orf41 protein. DRAQ5 was used as a nuclear marker. Overlapping of both signals (MERGE) is shown on the right.

### C15orf41 Localization Into Nuclear and Cytosolic Compartments

We first assessed the turnover and localization of the C15orf41 protein in Hek293 cells transiently transfected with pCMV-tag1-C15orf41. Time-course analysis showed a gradual increase of *C15orf41* gene expression in cells transfected with WT clone at 16, 24, and 48 h compared to those transfected with empty vector (EV) (Figure 3A). Conversely, WB analysis on the same harvested cells revealed a marked increase of C15orf41 protein level at 16 h after transfection, with a progressive decrease of the C15orf41-FLAG signal, which resulted highly down-regulated at 48 h after the transfection (Figure 3B).

To investigate C15orf41 localization, we assessed the endogenous protein levels and localization of the protein by both WB and IF on a nuclear and a cytosolic fraction of Hek-293 cells (Figure 3C,D). Both analyses confirmed that the protein was mainly expressed in the nucleus, but also in the cytosol compartment, even if in a smaller amount, suggesting a role of the protein in these two cellular compartments (Figure 3D and Supplementary Figure S3). No co-localization of C15orf41 with nucleoli was observed (Supplementary Figure S3).

**FIGURE 4:**
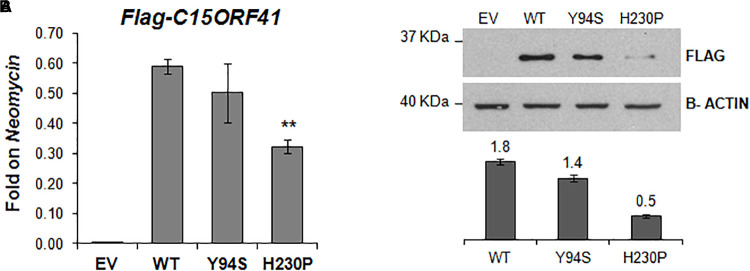
*In vitro* characterization of C15orf41 mutants. **(A)** The panel shows *FLAG-C15orf41* mRNA expression normalized on *Neomycin* of Hek-293 cells over-expressing pCMV-tag1-C15orf41-WT, -Y94S, and -H230P compared to those transfected with EV at 16 h after the transfection. *P* value by Student’s *t*-test. ^∗∗^*P* < 0.01. **(B)** The panel shows WB analysis of Hek-293 cells over-expressing pCMV-tag1-C15orf41-WT, -Y94S, and -H230P compared to those transfected with EV at 16 h after the transfection. The histogram shows the densitometric quantification based on the β-actin as a loading control. Data derived from three experiments are presented as mean ± SD. Sizes (in kDa) are on the left.

### Characterization of C15orf41-H230P and -Y94S Mutants

To study *in vitro* the pathogenetic effect of the two variants, we evaluated gene expression and protein level of both C15orf41-H230P and C15orf41-Y94S mutants at 16 h after transfection in Hek-293. In agreement with the *ex vivo* data on both patients, we observed a sharp decrease of both gene expression and protein levels in cells over-expressing C15orf41-H230P mutant compared to C15orf41-WT ones (Figure 4A,B and Supplementary Figure S1). Conversely, only a slight reduction in gene expression and protein level in cells over-expressing C15orf41-Y94S was observed (Figure 4A,B and Supplementary Figure S1).

**FIGURE 5:**
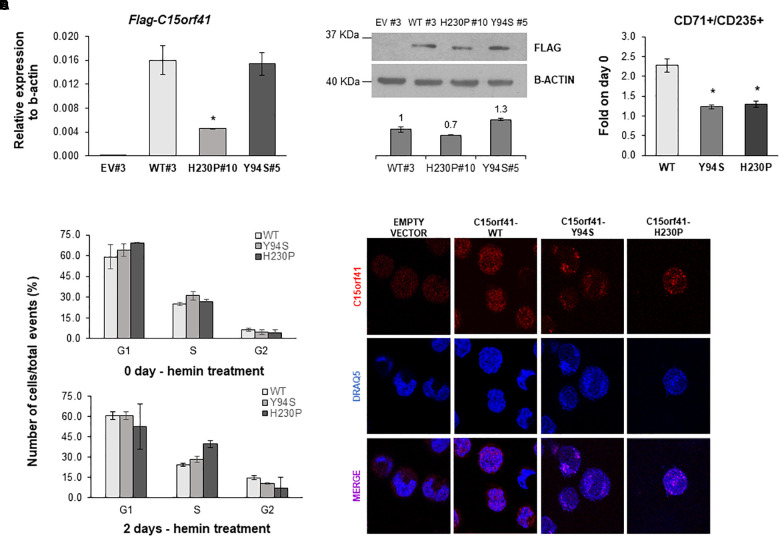
Analysis of K562-C15orf41 over-expressing clones during hemin-induced erythroid differentiation. **(A)** *FLAG-C15orf41* mRNA relative expression to β-actin of K562 cells stably over-expressing C15orf41-WT, -Y94S, and -H230P compared to the EV. Data derived from two experiments are presented as mean ± SD. *P* value by Student’s *t*-test. ^∗^*P* < 0.05. **(B)** WB analysis of K562 cells stably over-expressing C15orf41-WT, -H230P, and -Y94S compared to the EV. The histogram shows the densitometric quantification based on the β-actin. Data derived from two experiments are presented as mean ± SD. Sizes (in kDa) are on the left. **(C)** Erythroid differentiation markers of C15orf41-K562 stable clones. The histogram shows the percentage of CD71^+^/CD125^+^ cells at two days of hemin treatment normalized on untreated cells (0 days). Data derived from two experiments are presented as mean ± SD. *P* value by Student’s *t*-test. ^∗^*P* < 0.05. **(D)** The histograms show the number of K562 over-expressing pCMV-tag1-C15orf41-WT, -Y94S, and -H230P cells on total events (%) in G1, S, and G2 phases of the cell cycle. Data derived from two experiments are presented as mean ± SD. **(E)** Immunofluorescence analysis of K562 stable clones is shown. Rabbit anti-C15orf41 antibody was used to stain C15orf41 protein. DRAQ5 was used as a nuclear marker. Overlapping of both signals (MERGE) is shown at the bottom.

To obtain a reliable cellular model, able to be induced to erythroid differentiation, we developed K562 cells stably over-expressing C15orf41-WT and both mutants. Over-expressing clones were selected by measuring *Neomycin* relative gene expression in each K562 clone and comparing the digestion pattern of mutant vs. WT clones (Supplementary Figure S4). K562 selected clones WT#3 and Y94S#5 showed strong over-expression of *C15orf41* compared to those observed in K562 EV#3 clone. Instead, H230P#10 cells showed a marked gene down-regulation respect to WT#3 cells (Figure 5A). WB analysis confirmed the same trend for all the clones (Figure 5B).

To investigate if both mutants could affect erythroid differentiation, we treated K562 cells with hemin. Evaluation of CD71 and CD235 differentiation markers showed a statistically significant decreased percentage of CD71^+^/CD235^+^ cells in both Y94S and H230P clones compared to the WT one (Figure 5C). Moreover, we observed a slight increase of the rate of S-phase at cell cycle analysis in K562 cells over-expressing Y94S and H230P mutants compared to WT, although not statistically significant (Figure 5D and Supplementary Figure S5). To note, immunolocalization analysis of C15orf41 protein in K562 stable clones highlighted a preferential localization of the mutated proteins within nuclear compartment compared to WT one, similarly to those observed in Hek-293 cells transiently over-expressing C15orf41 mutants (Figure 5E).

## Discussion

CDA type I is an autosomal recessive disorder that belongs to the heterogeneous group of inherited BM failure syndromes. To date, two causative genes have been associated to this condition: *CDAN1* that is the most frequently mutated; *C15orf41* that has been found mutated in five unrelated patients, so far (Babbs et al., 2013; Palmblad et al., 2018; Russo et al., 2018). Most of the CDA I patients exhibit lifelong macrocytic anemia with variable values of Hb. *C15orf41* patients show clinical features like *CDAN1* ones. Anyhow, a slight difference in Hb level and MCV value has been observed between the two subgroups of patients (Gambale et al., 2016).

We herein described two unrelated cases of C15orf41-related CDA I. Both patients presented clinical characteristics, hematological status, and morphological features of erythroblasts compatible with a suspicion of CDA I. Particularly, the presence of a substantial amount of inter-nuclear bridges between erythroblasts, a typical feature of CDA I, at the BM analysis of the case 1 (A-II.2), prompted us to perform the molecular screening of both CDA I causative genes. No causative variants in *CDAN1* were identified, while genetic testing of *C15orf41* highlighted the presence of the homozygous missense mutation Y94S. This variant resulted annotated on public databases as ultra-rare single nucleotide variant. Of note, it is a novel missense change at an amino acid residue where a different pathogenic missense change, Y94C, has been previously described (Babbs et al., 2013). Case 2 (B-II.1) was initially suspected of suffering from CDA type II, since he presented normocytic anemia and non-specific morphological erythroblast features, such as the presence of bi- and multi-nuclearity, megaloblastic changes, but no inter-nuclear bridges. Of note, among syndromes showing dyserythropoiesis, there is not a full concordance between experienced hematologists in recognition of these features (Goasguen et al., 2018). Indeed, accurate molecular screening remains the most reliable diagnosis for these patients. First genetic testing for *SEC23B* revealed no mutations in this gene. Thus, the patient was analyzed by a t-NGS panel for red blood cell disorders, that allowed us the identification of the homozygous missense variant H230P in the *C15orf41* gene (Russo et al., 2018).

To investigate the expression and subcellular localization of C15orf41, we expressed the full-length WT protein fused to a FLAG-tag. Time-course analysis evidenced an indirect correlation between gene expression and protein levels, suggesting a rapid turnover of the protein. It was recently found that C15orf41 has at least three post-translational modification sites, such as K50 (Acetylation), T114 (Phosphorylation) and K176 (Ubiquitination) (Ahmed et al., 2018). Since that ubiquitination is one of the most common signals for proteasome-mediated degradation (Hershko and Ciechanover, 1998), we speculated that C15orf41 is degraded via proteasome during the cell cycle. Moreover, this data is corroborated by the fact that it could be a cell cycle-regulated protein, as well as Codanin-1 (Noy-Lotan et al., 2009), and that the two proteins could interact. Our *ex vivo* and *in vitro* analyses demonstrated that *C15orf41* and *CDAN1* gene expression levels were directly correlated in patients, healthy controls, and different cell lines. Of note, Codanin-1 was proved to be part of the cytosolic Asf1-H3-H4-importin-4 complex, which is implicated in nucleosome assembly and disassembly (Ask et al., 2012).

Similarly, C15orf41 was predicted to interact with Asf1b (Ewing et al., 2007). These data suggested that both proteins are needed together to accomplish their function, thus could be regulated by the same mechanism, could control each other in a positive feedback loop, or could interact with each other. To note, *CDAN1* and *C15orf41* are ubiquitously expressed genes, but their alterations mainly affect the erythroid lineage. One possible explanation may be that erythroid progenitors have a uniquely fast cell cycle, although CDA patients do not manifest abnormalities of other tissues containing fast-dividing cell types, such as gut epithelium or hair follicles. Other hypotheses include nuclear extrusion in erythroblasts, which requires the eviction of histones, such as H3 and H4, and C15orf41 and Codanin-1 may play a role in this process (Roy and Babbs, 2019).

The analysis of cytosolic and nuclear fractions demonstrated that C15orf41 endogenous protein exhibits mainly nuclear localization. Accordingly, nuclear localization signals and nuclear export signals were predicted in the amino acid sequence, confirming that the protein is exported from the nucleus to the cytoplasm and vice-versa. Once again, changes in C15orf41 nuclear-cytoplasmic localization could represent a mechanism, or the effect, of its regulation. According to the predicted Holliday junction resolvase function of C15ORF41 and its potential role in DNA repair machinery as guardians of genome integrity and viability, we initially hypothesized that C15orf41 could localize in the nucleoli. Indeed, it was recently demonstrated that the nucleolus, long regarded as a mere ribosome producing factory, plays a crucial role in monitoring and responding to cellular stress, as well as in DNA repair mechanisms (Mayer and Grummt, 2005; Ogawa and Baserga, 2017). However, our immunolocalization data did not support this hypothesis.

We further characterized the identified variants by both *ex vivo* and *in vitro* functional analyses. The two mutations showed different behavior. Indeed, the Y94S variation did not affect gene expression, and only slightly decreased the protein level. On the contrary, the H230P mutation induced a sharp decrease in gene expression and protein level.

Of note, Y94S variant is located in the two turn-helix-turn DNA binding domains (DBD) of the protein, together with the previously identified Y94C and P20R mutations (Babbs et al., 2013; Palmblad et al., 2018). On the contrary, H230P variant is located in the PD-(D/E) XK nuclease domain, as well as the two causative mutations L178Q and Y238C (Babbs et al., 2013; Palmblad et al., 2018). Therefore, we might assume that these variants could have a different effect on both the protein function and the pathogenetic mechanism of the disease.

Since no impaired expression of C15orf41-Y94S was observed, we speculate that this mutation could affect the three-dimensional structure of the protein and, thus, undermine the binding to the DNA.

Since CDA I mutated proteins affect mainly the erythroid lineage, we developed K562 cells stably over-expressing C15orf41 WT and mutants to induce erythroid differentiation. This cellular model allowed us to demonstrate that both mutant clones showed impaired erythroid differentiation, exhibiting a decreased percentage of CD71^+^/CD235^+^ cells at two days of hemin treatment. Moreover, both Y94S and H230P clones were retained in the S phase of the cell cycle during differentiation, although with a different degree. It has been already demonstrated that there is an interdependence between S-phase progression and an essential commitment step during erythroid differentiation in which, within few hours, cells become dependent on the hormone erythropoietin, undergo activating changes in chromatin of red cell genes, and activate GATA-1, the erythroid master transcriptional regulator. Arresting S-phase progression at this time prevents the execution of this commitment step and subsequent induction of red cell genes (Pop et al., 2010). Of note, *CDAN1*-CDA Ia cultured erythroblasts showed an increase in S-phase cells, suggesting a cell cycle arrest (Tamary et al., 1996). Nevertheless, based on the present data, we are not able to establish if the increased number of cells in S-phase represents faster cycling cells or a block in S-phase.

This study represents the first investigation of both the expression and the localization of C15orf41. Our *ex vivo* and *in vitro* analyses demonstrated that C15orf41 and CDAN1 are tightly correlated, suggesting a shared mechanism of regulation between the two genes and related proteins. The different behavior of both Y94S-DBD-mutation and H230P-PD-(D/E) XK-mutation could be related to the dual function of the C15orf41 protein within separate subcellular compartments. Nevertheless, both variants resulted in impaired erythroid maturation, suggesting the block of cell cycle dynamics as a putative pathogenic mechanism for C15orf41-related CDA I.

## Ethics Statement

Ethical UC committee University Federico II of Naples, n. 252/18.

## Author Contributions

RR, RM, and IA designed and conducted the study, and prepared the manuscript. AI critically reviewed the study. GDR and BR collaborated to the generation of cellular models. FM performed Sanger sequencing and t-NGS. MR performed flow cytometry analyses. SU, SB, and AG cared for the patients.

## Conflict of Interest Statement

The authors declare that the research was conducted in the absence of any commercial or financial relationships that could be construed as a potential conflict of interest.

## Footnotes

1 http://wintervar.wglab.org/

2 http://r2.amc.nl
