# Corrigendum: Characterization of Two Cases of Congenital Dyserythropoietic Anemia Type I Shed Light on the Uncharacterized C15orf41 Protein

**DOI:** 10.3389/fphys.2020.00940

**Published:** 2020-08-11

**Authors:** Roberta Russo, Roberta Marra, Immacolata Andolfo, Gianluca De Rosa, Barbara Eleni Rosato, Francesco Manna, Antonella Gambale, Maddalena Raia, Sule Unal, Susanna Barella, Achille Iolascon

**Affiliations:** ^1^Dipartimento di Medicina Molecolare e Biotecnologie Mediche, Università degli Studi di Napoli Federico II, Naples, Italy; ^2^CEINGE Biotecnologie Avanzate, Naples, Italy; ^3^Division of Pediatric Hematology, Hacettepe University, Ankara, Turkey; ^4^SSD Talassemie, Anemie Rare e Dismetabolismi del Ferro, Ospedale Pediatrico Microcitemico Antonio Cao, Azienda Ospedaliera Brotzu, Cagliari, Italy

**Keywords:** CDA (I–III), *C15ORF41*, functional characterization of proteins, genetic testing, anemia

In the published article, there was an error in affiliation 3. Instead of “SSD Talassemie, Anemie Rare e Dismetabolismi del Ferro, Ospedale Pediatrico Microcitemico Antonio Cao, Azienda Ospedaliera Brotzu, Cagliari, Italy”, it should be “Division of Pediatric Hematology, Hacettepe University, Ankara, Turkey”.

The authors apologize for this error and state that this does not change the scientific conclusions of the article in any way. The original article has been updated.

