# Supplementary material for: Characterization of Two Cases of Congenital Dyserythropoietic Anemia Type I Shed Light on the Uncharacterized C15orf41 Protein
Source: Front Physiol. 2019 May 22;10:621. doi: 10.3389/fphys.2019.00621 (PMC6539198; doi:10.3389/fphys.2019.00621)
Supplement: Supplementary file 1 [file Data_Sheet_1.PDF]

SUPPLEMENTAL DATA

Article title:

Characterization of two cases of congenital dyserythropoietic anemia type I shed light on the uncharacterized C15orf41 protein

Authors:

Russo Roberta, Roberta Marra, Immacolata Andolfo, Gianluca De Rosa, Barbara Eleni Rosato, Francesco Manna, Antonella Gambale, Maddalena Raia, Sule Unal, Susanna Barella, Iolascon Achille

Corresponding author:

Roberta Russo, Dipartimento di Medicina Molecolare e Biotecnologie Mediche, Università degli Studi di Napoli Federico II, Napoli, Italy; CEINGE Biotecnologie Avanzate, Napoli, Italy; roberta.russo@unina.it

Table of contents:

- Methods
- Figure S1
- Figure S2
- Figure S3
- Figure S4
- Figure S5

Methods

Quantitative real-time PCR analysis

Quantitative RT-PCR (qRT-PCR) using Power SYBR Green PCR Master Mix (Applied Biosystems) was performed to evaluate the gene expression of *Neomycin* resistance gene (expressed by pCMV-Tag1 vector) that was used as a control of transfection efficiency for K562 stable clones. *β-actin* was used as internal control. Relative gene expression was calculated by using the $2^{-\Delta Ct}$ method.

K562 stable clones

For K562 stably over-expressing FLAG-C15orf41 gene, 10^6 cells were transfected with pCMV-Tag1-C15orf41 plasmids (2µg), using Hily Max DNA Transfection Reagent (Dojindo Laboratories). After 48 hours, G418 (0.6 mg/mL) was added as a selection marker. Clones were generated by plating on a 48-well an appropriate number of cells and diluting them to decrease the cell number (limiting dilution method) progressively. Resistant clones were expanded, and then only the FLAG-C15orf41 over-expressing clones were selected for the following experiments. C15orf41 WT and mutant clones were also confirmed using the restriction analysis, as predicted by Restriction of DNA sequences Tool (http://insilico.ehu.eus/restriction/two_seq/index.php, freeware online).

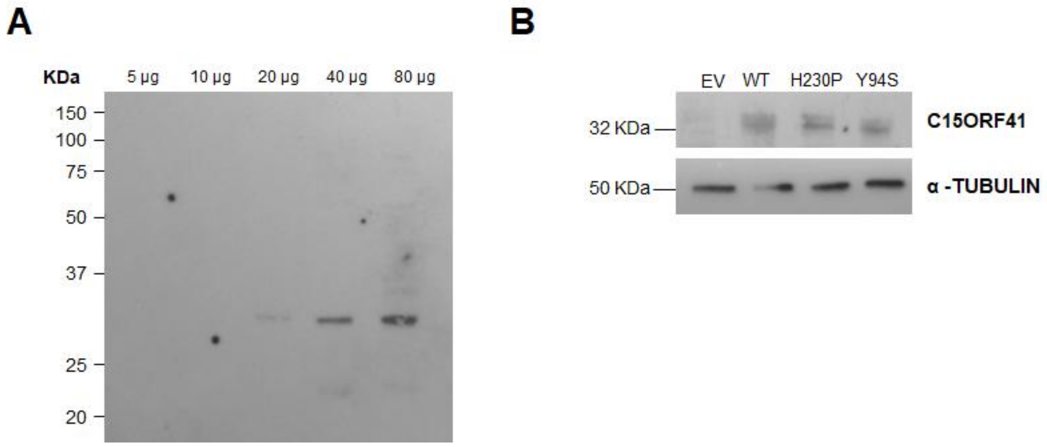

Figure S1. Evaluation of the specificity of anti-C15orf41 antibody (Atlas Antibodies HPA061023). (A) Dilution analysis of C15orf41 wild-type in stably over-expressing K562 cells is shown. To note, the predicted molecular weight of C15orf41 is 32.264 KDa (Uniprot ID: Q9Y2V0). **(B)** WB on cytosolic fraction of Hek-293 cells transiently over-expressing pCMV-tag1-C15orf41-WT, -Y94S, and -H230P; α-TUBULIN is the loading control.

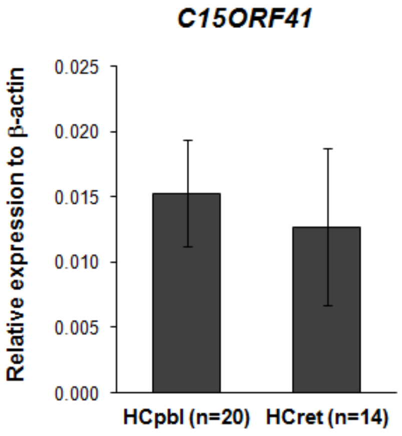

Figure S2. Analysis of C15orf41 gene expression in peripheral blood leukocytes and reticulocytes. *C15orf41* mRNA relative expression to *β-actin* in both peripheral blood leukocytes (pbl) and reticulocytes (ret) from healthy controls (HC) is shown. Data are presented as mean ± SE.

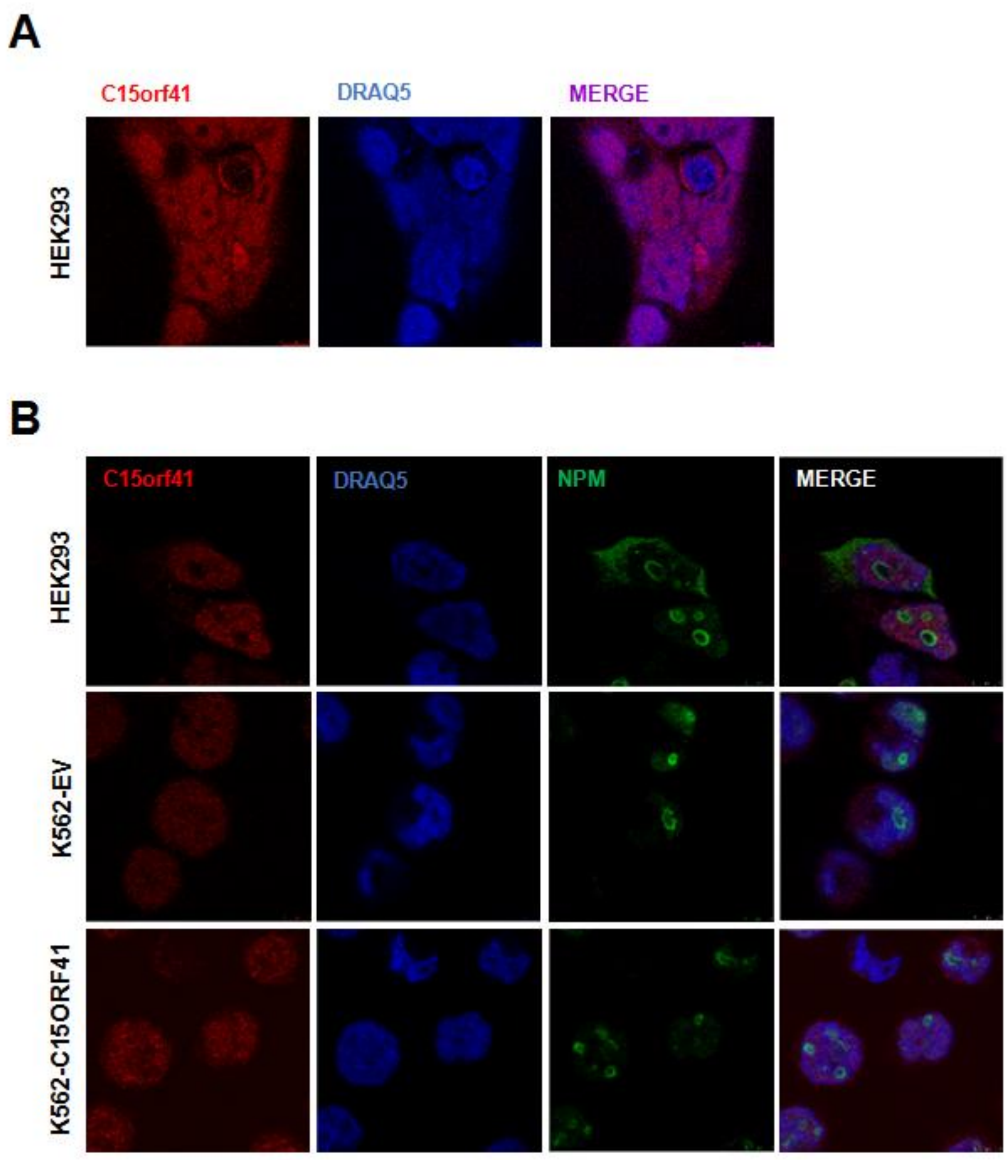

Figure S3. Analysis of the subcellular localization of the C15ORF41 protein. (A) Immunofluorescence analysis of Hek-293 is shown. Rabbit anti-C15orf41 antibody was used to stain C15orf41 protein. DRAQ5 was used as a nuclear marker. Overlapping of both signals (MERGE) is shown on the right. **(B)** Immunofluorescence analysis of Hek-293 and K562 cells is shown. Rabbit anti-C15orf41 antibody was used to stain C15orf41 protein. DRAQ5 was used as a nuclear marker. Nucleophosmin (NPM) was used as nucleolar marker. Overlapping of both signals (MERGE) is shown on the right.

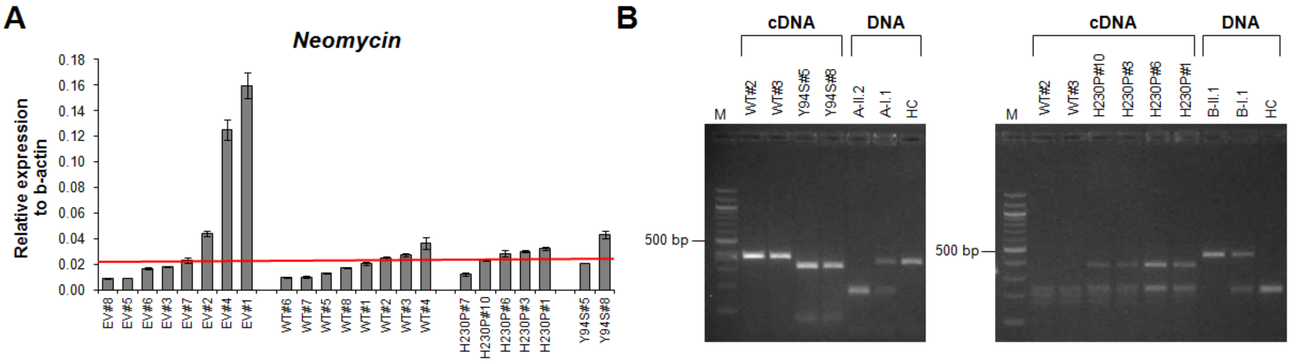

Figure S4. Selection of K562 stable clones for C15ORF41-WT, -Y94S, and -H230P. (A) *Neomycin* relative expression to *b-actin* of K562 clones over-expressing pCMV-tag1-C15ORF41-WT, pCMV-tag1-C15ORF41-H230P, and pCMV-tag1-C15ORF41-Y94S. Data are presented as mean ± SD of three replicates. Clones that showed similar expression levels, whose levels are along with the median value of *Neomycin* expression (red line), underwent to further analysis. **(B)** On the left, digestion pattern of C15ORF41-Y94S over-expressing clones and family A carrying the Y94S variant by HinfI enzyme. The Y94S variant creates a new restriction site for HinfI enzyme, generating a 298 bp-fragment at cDNA level and a 164 bp-fragment at DNA level, while no restriction products are obtained in the presence of WT genotype. On the right, digestion pattern of C15ORF41-H230P over-expressing clones and family B, carrying the H230P variant, by FatI enzyme. The H230P variant abrogates the restriction site for FatI enzyme, generating the 357 bp- and 222 bp-fragments at cDNA level, while no restriction products were obtained in the presence of the mutated base at DNA level. M=marker 100bp; HC, healthy control.

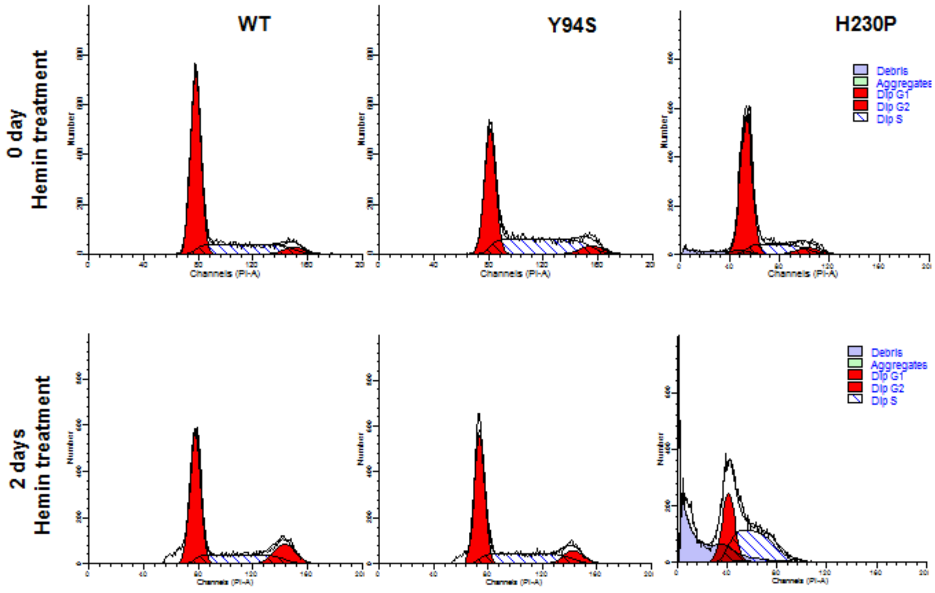

Figure S5. Cell cycle analysis. Cell cycle distribution of C15orf41-K562 stable clones at 0 and two days of hemin treatment. Cell cycle was analyzed using FACS analysis of propidium iodide-stained cells.
